# Maxillary sinusitis developed as sequelae of accidental middle turbinectomy that occurred during nasotracheal intubation: a case report

**DOI:** 10.1186/s12871-021-01344-3

**Published:** 2021-04-22

**Authors:** Joungmin Kim, Taehee Pyeon, Hyun Jung Lee, Hyung Chae Yang

**Affiliations:** 1 Department of Anesthesiology and Pain Medicine, Chonnam National University Medical School and Chonnam National University Hospital, 5 Hak-dong, Gwangju, 501-746 Republic of Korea; 2 Department of Otolaryngology-Head and Neck Surgery, Chonnam National University Medical School and Chonnam National University Hospital, Gwangju, South Korea

**Keywords:** Accidental turbinectomy, Airway obstruction, Nasal septal deviation, Nasotracheal intubation, Complication

## Abstract

**Background:**

Nasotracheal intubation is a very useful technique for orofacial or dental surgery. However, the technique itself can be more traumatic than that of orotracheal intubation. Complications such as turbinectomy or bleeding are often reported. However, little is known about the follow-up of patients after these complications.

**Case presentation:**

The present case describes an accidental middle turbinectomy that led to endotracheal tube obstruction during nasotracheal intubation, and discusses its long-term follow-up. A 19-year-old man underwent mandibular surgery under general anesthesia and nasotracheal intubation. His right middle turbinate was completely avulsed and became firmly occluded within the tube during nasotracheal intubation. The nasotracheal intubation was performed again and the operation was completed safely. The patient was discharged without sequelae after postoperative care. However, he had symptoms of nasal obstruction and sleep disturbance for 3 months postoperatively. Synechiae were detected between the nasal septum and lateral nasal wall on a right rhinoscopic examination and facial computed tomography at 3 months postoperatively. Additionally, he showed ipsilateral maxillary sinusitis on facial computed tomography at the 2-year follow-up examination.

**Conclusions:**

Nasotracheal intubation can cause late complications as well as early complications. Therefore, if nasotracheal intubation is to be performed, the anesthesiologist should identify the nasal anatomy of the patient accurately and prepare appropriately. In addition, if complications occur, follow-up observation should be performed.

## Background

The technique of nasotracheal intubation was first described by Kuhn in 1902 [1]. Subsequently, this technique has gained popularity in the induction of anesthesia for intraoral, pharyngeal, and laryngeal surgeries, and for improved visualization during neck surgeries [2]. However, the technique itself is more traumatic than orotracheal intubation. Factors that contribute to this trauma include practitioner inexperience and lack of supervision, as well as patient anatomical variations, past reconstructive surgery in the orofacial area, and cleft palate or facial bone fracture, which can disrupt the passage of the nasotracheal tube [3–5].

Complications of nasotracheal intubation include epistaxis, traumatic avulsion of the nasal cavity structure or the nasopharynx, and bacteremia caused by the abrasion of nasal mucosa or movement of nasal flora into the trachea [2]. Anatomical variations in the nasal cavity (e.g., septal deviation or hypertrophy of the turbinate) are common and can be asymptomatic in patients [5, 6]. Thus, a careful assessment of the nasal cavity is needed both before nasotracheal intubation and before orotracheal intubation. However, not all methods for prediction of the appropriate nostril to intubate are beneficial [6]. Here, we describe a patient who underwent traumatic middle turbinectomy that was complicated by endotracheal tube obstruction during nasotracheal intubation and developed ipsilateral maxillary sinusitis. To the best of our knowledge, this is the first case report of long-term follow-up in a patient with traumatic turbinectomy complicated by nasotracheal intubation. Informed consent was obtained from the patient for this clinical case report.

## Case presentation

**Fig. 1:**
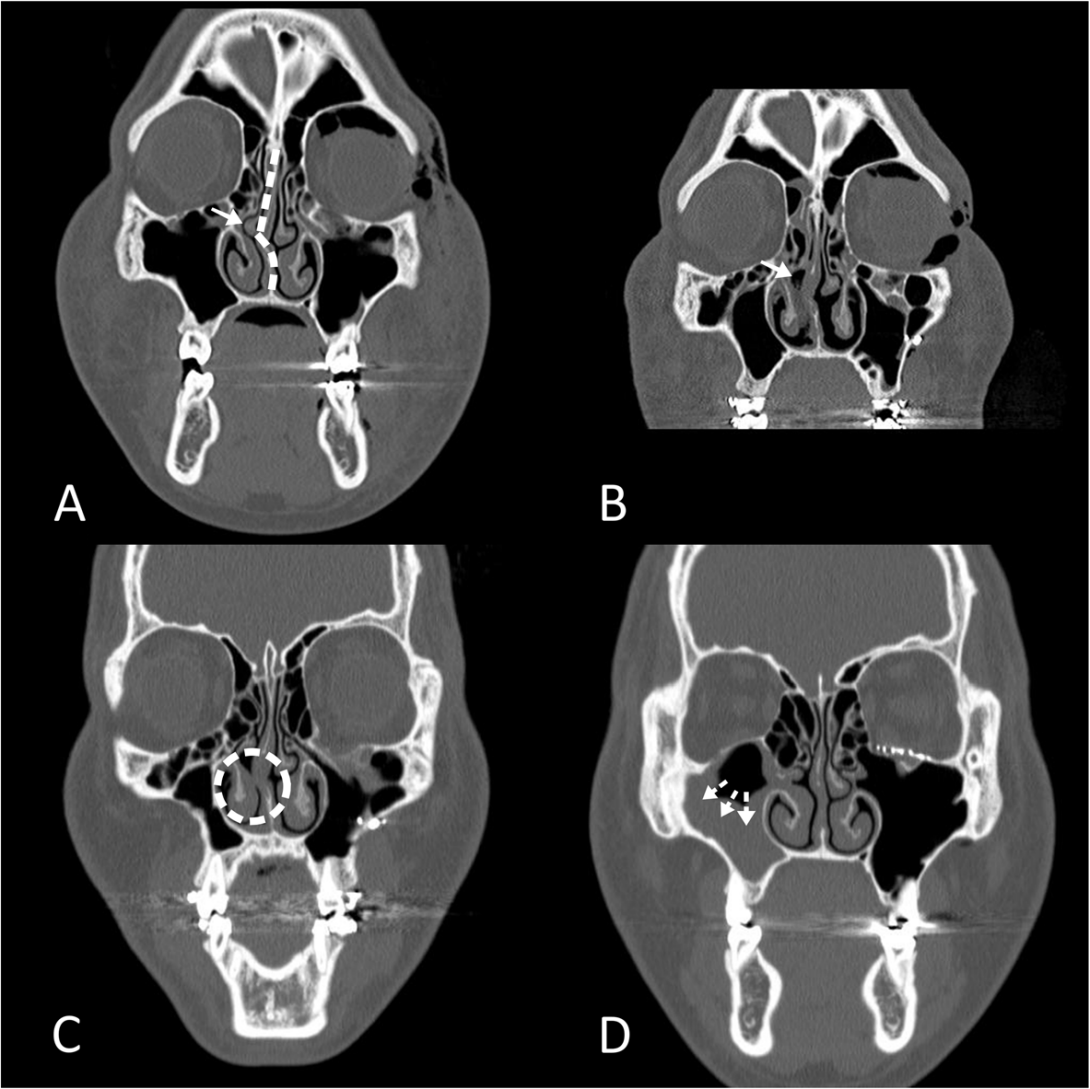
Facial computed tomography (CT) scans of the nose. **a** Preoperative CT scan; right deviated nasal septum is noted (dotted line). **b** One-day postoperative CT scan; absence of right middle turbinate is noted (White arrow on panel A and B). **c** Three-month postoperative CT scan; formation of synechiae is noted in right nasal cavity (dotted circle). **d** Two-year postoperative CT scan; right maxillary sinusitis is noted (dotted arrow).

A 19-year-old man was transferred to the emergency department at the Chonnam National University Hospital because of painful swelling of the left mandibular area due to a bicycle accident that had occurred 12 hours prior. His medical history was nonspecific except for a fatty liver. A physical examination revealed stable vital signs but he was in acute distress. Head and neck examination indicated severe tenderness and swelling over the left mandibular angle and left periorbital regions. A CT scan and clinical examination showed that the patient had sustained a left mandibular angle fracture and left infraorbital wall fracture, requiring surgical intervention. Open reduction of the mandibular angle fracture was planned at the Department of Oral and Maxillofacial Surgery; the surgery was planned to include general anesthesia with nasotracheal intubation. Informed consent was obtained from the patient for the surgical procedure. The results of pre-anesthesia evaluation, including routine investigations (e.g., chest X-ray, electrocardiography, and blood chemistry), were within normal limits. The patient was classified as American Society of Anesthesiologists physical status II. He had no unusual findings on upper airway examination; however, he exhibited restricted mouth opening, presumably due to pain. The nasal septum deviated slightly to the right; mucosal edema on both sides of the nasal cavity was evident on preoperative facial CT (Fig. 1a). At the preoperative visit, the patient reported easier breathing through the right side of his nose on the nasal obstruction test.

Standard monitoring (e.g., electrocardiography, oxygen saturation by pulse oximetry, noninvasive sphygmomanometry, and capnography) were placed upon arrival in the operating room. Anesthesia was induced with 1 μg/kg intravenous remifentanil and 2 mg/kg intravenous propofol, followed by 0.5 mg/kg intravenous rocuronium after full preoxygenation. When neuromuscular blockade was achieved, a 7.0-mm cuffed nasal preformed endotracheal tube (Portex®; Smiths Medical International, Hythe, UK), lubricated with 2% xylocaine jelly, was inserted into the right nares with moderate resistance. There was resistance in the attempt to enter to the right, so we attempted to enter again through the left nostril, but gave up due to greater resistance. Advancement of the tube through the right nostril, with the bevel of the tube facing medially, was ultimately achieved into the hypopharynx. The tube was further inserted into the trachea without difficulty with the assistance of McGill forceps under direct visualization via laryngoscopy. Manual ventilation was initiated with 100% oxygen; however, airway pressure increased to 40–50 cmH_2_O, and capnography revealed a wider Q angle and steeper slope of the alveolar plateau, indicating airway obstruction. An endotracheal tube obstruction was regarded as the probable cause; an unsuccessful attempt was made to pass a suction catheter through the lumen of the endotracheal tube. The nasotracheal tube was removed and replaced with a new 7.0-mm nasotracheal tube through the right nostril again. After the tube had been replaced, intubation was confirmed with bilateral equal breath sounds, good chest excursions, and capnography assessment. Inspection of the removed tracheal tube revealed a 4-cm mass partially occluding the tube. The mass was identified as a turbinate, and the Department of Otorhinolaryngology was consulted. Open reduction and internal fixation of the mandibular fracture and orbital wall reconstruction using Medpor® were performed. After completion of the surgical procedure, the patient was extubated uneventfully in the operating room and transferred to the recovery room. There was no evidence of gross hemorrhage from the nose or the oral cavity.

**Fig. 2:**
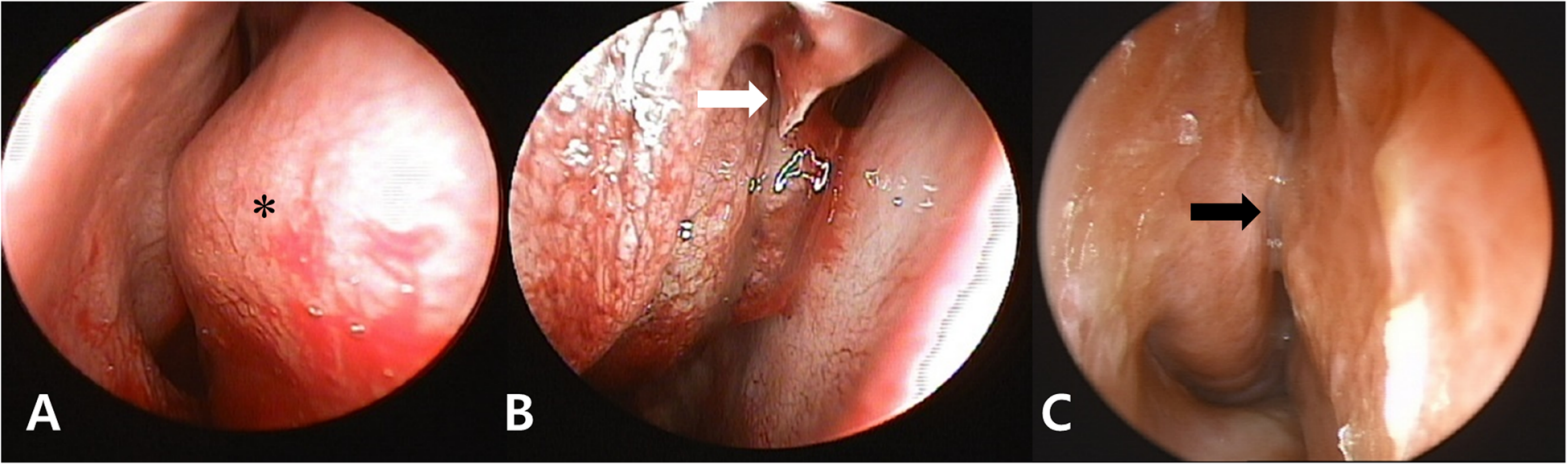
Nasal endoscopy findings in the right nasal cavity. **a** A right deviated nasal septum (*) and residual bloody spots, as well as **b** a small remnant of the middle turbinate, are present at 1 day postoperatively. **c** Formation of synechiae was observed at 3 months postoperatively.

An otolaryngologist performed a bilateral endoscopic nasal cavity examination on 1 day postoperatively. Inspection of the right nares revealed that the septum deviated to the right, especially at the level of the uncinate process; residual bloody spots (Fig. 2a) and the stalk remnants of the middle turbinate (Fig. 2b) were observed. The maxillary crest was wide and the mucosa was damaged. A tilted vomer and perpendicular ethmoid plate made an acute angle. However, there was no bleeding or leakage of cerebrospinal fluid. No endoscopic cauterization of the remaining right middle turbinate stalk or nasal packing was required to achieve hemostasis. Inspection of left nares also revealed mucosal damage. A facial CT scan taken 1 day postoperatively revealed remnants of the right middle turbinate stalk (Fig. 1b). The patient was followed up at the Department of Otorhinolaryngology before discharge, but he denied any nasal complications, bleeding, or obstruction.

At the 3-month postoperative follow-up, the patient had symptoms and signs of nasal obstruction in the traumatized nasal cavity; he also reported sleep disturbance. Ipsilateral synechiae had formed between the nasal septum and lateral nasal wall, as determined by a right rhinoscopic examination (Fig. 2c) and facial CT (Fig. 1c). A home sleep test was performed to determine the degree of sleep apnea, which revealed a respiratory disturbance index of 11.3 events per hour. The synechiae resolved rapidly after simple lysis with concurrent septoplasty, and the signs of nasal obstruction and sleep disturbance were much improved. The patient remained recurrence-free beyond simple lysis of the synechia for the next 65 months.

At the 2-year follow-up, the patient exhibited nasal fullness and a yellowish discharge. Although right rhinoscopy indicated that no new synechiae had formed, ipsilateral maxillary sinusitis was observed on facial CT (Fig. 1d). The symptoms of maxillary sinusitis improved after medical treatment.

## Discussion

We have described a patient in whom accidental middle turbinectomy caused endotracheal tube obstruction during nasotracheal intubation. The patient developed synechiae between the nasal septum and lateral nasal wall, accompanied by symptoms and signs of nasal obstruction and sleep disturbance; he underwent a lysis operation at 3 months postoperatively. At the 2-year follow-up, he showed ipsilateral maxillary sinusitis with nasal fullness and a yellowish nasal discharge, which improved after medical treatment.

Nasotracheal intubation is an effective means of securing the airway while preserving the surgical field during oral or maxillofacial surgery. The tube must be passed through the narrow and long nasal cavity without securing a direct view; more complications can thus occur, compared with orotracheal intubation [7]. During nasotracheal intubation, the endotracheal tube in the nostril may pass through either the upper pathway between the middle and inferior turbinates, or through the lower pathway between the inferior turbinate and the floor of the nasal cavity [8]. The lower pathway is considered safer and is thus the preferred route, because it is located at a greater distance from the middle turbinate and cribriform plate.

The tube is typically inserted perpendicularly to the facial plane in an anteroposterior direction, rather than forcible insertion superiorly; thus, the tube is passed through the lower pathway between the inferior turbinate and the floor of the nasal cavity [9]. Because of its location, the inferior turbinate is the most frequently injured nasal structure during intubation through the nasal cavity. However, in patients with hypertrophy of the inferior turbinate, edema of the inferior turbinate mucosa, or a spur in the nasal septum, the middle turbinate is located in front of the skull base; thus, it protrudes into the nasal cavity, which increases the possibility of damage during intubation [10]. However, it is difficult to direct a preformed nasotracheal tube along the floor of the nasal cavity, as these tubes can travel in the cephalad direction, despite specific efforts to avoid this route [8]. Overall, in the presence of the curved nasal septum at the beginning of entry, a preformed nasotracheal tube may need to be passed through the very narrow upper pathway; thus, insertion of the tube despite resistance may cause injury to the middle turbinate, as in our patient.

Complications of nasotracheal intubation include nasal bleeding, bacteremia, partial or complete obstruction of the tube, and rarely, permanent damage to intranasal structures has been reported [9]. In this case, the blind intubation technique was used when performing nasotracheal intubation. The blind procedure has a risk of inflicting great harm to the patient even if it proceeds without problems [11]. In particular, caution should be taken to patients with facial fractures because there is a possibility of a concurrent skull base fracture.

To reduce these complications, the use of topical vasoconstrictor agents (e.g., cocaine, epinephrine, xylometazoline, oxymetazoline, lidocaine-phenylephrine combination, or 10% lidocaine) to contract the turbinate mucosa [12], as well as lubrication and pre-softening of the tube [13], use of a small tube [9], and selection of bevel direction [14], have been recommended. An anesthesiologist may choose a smaller tube. For example, a 7.0 mm tube can be used for adult males and 6.0 mm for adult females. Another way to reduce intranasal damage is to use a urethral catheter [15] or nasogastric tube [16] to guide an endotracheal tube to pass through a narrow nasal cavity. If the tube fails to advance, the intubator can use the fiberscope or manipulate the tube (cephalad distortion of the tube, anti-clockwise rotation of level). However, no excessive force should be given to insert.

In addition, rhinoscopy [17], facial CT [18], flowmetry [19], and a nasal obstruction test have been used to select the proper nostril for tube insertion. Among these methods, the nasal obstruction test is an easy method to use in clinical practice; it involves alternating nostril blocking during breathing and identification of the less obstructed naris. Our patient had been intubated through the right nostril, although this method is less reliable than rhinoscopy [6].

The middle turbinate is located in the anterior skull base adjacent to the cribriform plate, and is attached loosely to the ethmoid bone posteriorly. Thus, there is a risk of greater complications in patient with trauma to the middle turbinate, including those with massive epistaxis, cerebrospinal fluid rhinorrhea, or olfactory nerve damage [20]. When a diagnosis has been established regarding accidental avulsion of a nasal structure occluding the tube, intubation through the traumatized nares is recommended to produce a tamponade effect and minimize bleeding from the affected nares [20]. The patient was re-intubated through the same nostril, rather than in the opposite nostril [20], which resulted in minimal bleeding from the injured turbinate. Indeed, neither endoscopic cauterization of the turbinate nor nasal packing was required to produce hemostasis in our patient.

Nevertheless, traumatic removal of the middle turbinate mucosa resulted in the formation of synechiae causing signs and symptoms of nasal obstruction and sleep disturbance at 3 months postoperatively. Synechiae have been reported to form early during the postoperative period and resolve with simple lysis in patients who have undergone turbinectomy [21]. Thus, close follow-up during the initial postoperative stages is generally recommended to avoid long-term complications. Indeed, the symptoms and signs of nasal obstruction and sleep disturbance improved in our patient, following simple lysis with concurrent septoplasty. Our patient remained free of synechia for the next 65 months.

Ipsilateral maxillary sinusitis was observed on facial CT performed at 2 years postoperatively in our patient. Maxillary sinusitis has been described as a complication of prolonged nasotracheal intubation [22, 23]. An ostial obstruction has been causally related to the development of sinusitis in animal studies [24, 25]. This may have been applicable in our patient; notably, he did not exhibit a common cause of maxillary sinusitis (e.g., fungal ball or periapical abscess [26]). Another explanation is that impaired sinus mucociliary movement due to loss of the middle turbinate may have led to the development of maxillary sinusitis. This speculation is supported by prior findings that the middle turbinate is the primary site for impaction of inhaled particles and serves to protect the maxillary and ethmoid sinuses from inhaled air and the associated drying effects on sinus mucociliary clearance [27]; moreover, the middle turbinate plays an indirect role in sinus mucociliary clearance by blocking turbulent flow in the middle meatus [28].

In conclusion, in patients with trauma to the middle turbinate complicated by nasotracheal intubation, both early complications (e.g., life-threatening airway obstruction or bleeding) and late complications (e.g., nasal synechiae or sinusitis) can occur. If an anesthesiologist must perform nasotracheal intubation in a patient with septal deviation, sufficient preoperative nasal examination and preparation to reduce damage should be performed to avoid possible complications.

## Data Availability

Not applicable.

## Abbreviations

CT: Computed Tomography
